# Screening for Coping Style Increases the Power of Gene Expression Studies

**DOI:** 10.1371/journal.pone.0005314

**Published:** 2009-04-23

**Authors:** Simon MacKenzie, Laia Ribas, Maciej Pilarczyk, Davinia Morera Capdevila, Sunil Kadri, Felicity A. Huntingford

**Affiliations:** 1 Unitat de Fisiologia Animal, Department de Biología Cel.lular, Fisiologia i Immunologia, Facultat de Biociencies, Universitat Autonoma de Barcelona, Barcelona, Spain; 2 Polska Akademia Nauk, Zakład Ichtiobiologii i Gospodarki Rybackiej, Zaborze, ul. Kalinowa , Chybie, Poland; 3 Faculty of Biomedical & Life Sciences, Environmental & Evolutionary Biology, University of Glasgow, Glasgow, Scotland, United Kingdom; University of Lethbridge, Canada

## Abstract

**Background:**

Individuals of many vertebrate species show different stress coping styles and these have a striking influence on how gene expression shifts in response to a variety of challenges.

**Principal Findings:**

This is clearly illustrated by a study in which common carp displaying behavioural predictors of different coping styles (characterised by a proactive, adrenaline-based or a reactive, cortisol-based response) were subjected to inflammatory challenge and specific gene transcripts measured in individual brains. Proactive and reactive fish differed in baseline gene expression and also showed diametrically opposite responses to the challenge for 80% of the genes investigated.

**Significance:**

Incorporating coping style as an explanatory variable can account for some the unexplained variation that is common in gene expression studies, can uncover important effects that would otherwise have passed unnoticed and greatly enhances the interpretive value of gene expression data.

## Introduction

Studying changes in gene expression as individual organisms respond to environmental change is an invaluable tool in elucidating the mechanisms that determine the impact of such change on fitness. Measurement of gene expression has been greatly facilitated by the increasing availability of transcriptomic technologies. These have led to a rapidly expanding body of research addressing adaptive changes in gene expression in natural populations in various animal groups, including fish [1]–[4]. Such studies have tremendous potential for characterising in broad terms how patterns of gene expression change in response to challenge. However, questions have been raised about how differences at the transcriptome level are related to adaptive phenotypic variation [5].

We draw attention here to the fact that striking naturally-occurring differences in response to environmental change exist and argue that taking these into account can help to link events at the genetic and phenotypic level. For example, a study of gene expression in the hearts of individual male fish (*Fundulus heteroclitus*) provided with different energy substrates found significant, consistent individual variability in the metabolic use of the substrate, in mRNA expression and genes associated with substrate-specific metabolism [4]. Clustering of individual fish, *a posteriori*, on the basis of their gene expression profiles identified 3 distinct groups of individuals, with 80% of the reported variation being explained by grouping specific genes into relevant metabolic pathways. Significant differences in tissue-specific gene expression between populations were reported in the same fish species collected from different areas [6]. In both cases, variation in gene expression seems to be related to differences in the physiological status of individuals and the ecological context of populations.

Another potential source of individual variation in gene expression lies in differences in stress coping style (sometimes referred to as differences in temperament) shown by many species of animals. In a wide range of vertebrates, from monkeys, mice, rats to great tits and rainbow trout, striking and consistent individual variability in physiological and behavioural responses to challenge has been reported within animals of the same species, population, gender and age [7]. In ecological terms, it has been suggested that proactive animals will best flourish in stable, resource-rich environments at high population densities, while their reactive conspecifics flourish will at low densities, where resources are sparse and unpredictable [8]. Such syndromes of physiological and behavioural traits have been described in numerous homeothermic vertebrates [9]–[15] and also in fish [16]–[20].

In the present study, we demonstrate that such differences in coping styles can have major effects on levels of gene expression, both at baseline and in response to a bacterial lipolysaccharide (LPS) challenge. We have used an inflammatory challenge, intraperitoneal LPS, as this challenge causes a significant reorganization of the immune system and the subsequent transcriptomic remodeling in relevant subsets of cells is considered as one of the strongest reorganization events in physiological systems [21]. To our knowledge, coping style has not been considered as an explanatory variable in the majority of studies aimed at exploring the regulation of gene expression in animals responding to environmental change. The data presented here show that taking account of coping style facilitates the interpretation of gene expression studies, making it easier to relate events at the transcriptome level to adaptive phenotypic change.

## Results

**Figure 1:**
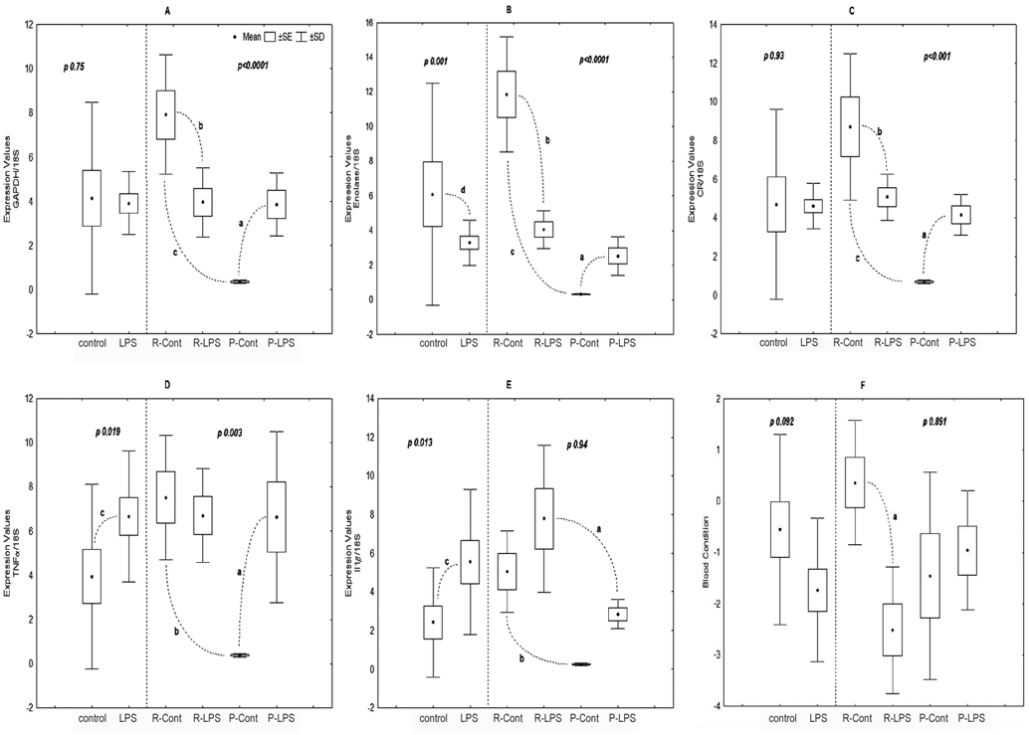
Gene expression in the brain of carp, C.carpio, screened for bold and timid behaviour and held under normoxic conditions under a natural photoperiod with water temperature of 20°C. After 8 weeks, individual fish from bold and timid groups were either injected intra-peroniteally with 6 mg/Kg of bacterial lipopolysaccharide, n = 6, or an equivalent volume of vehicle, PBS, n = 6. Each graph is represented in two parts, the left section shows the analysis without behavioural discrimination (ND), n = 12 and the right section displays results with discrimination (D), n = 6, where R and P represent reactive and proactive respectively. All data were analysed with a Factorial ANOVA. Letters represent p-values (post-hoc; Tukeys HSD) between groups. Blood condition scores were derived from a principal components analysis of hemoglobin concentration (Hb), hematocrit (Ht) and red blood cell count (RBC). The component accounted for 73% of the total variance with loading of 0.61 for Hb, 0.5 for Ht and 0.58 for RBC. A. GAPDH, ND p = 0.75, D p = 0.75, F (1,19) = 26.79, a. p = 0.016, b. p = 0.004, c. p = 0.0002. B. Enolase, ND p<0.001, D p = 0.000002, F (1,20) = 44.99, a. p = 0.000176, b. p = 0.000175. C Cortisol receptor (CR), ND p = 0.93, D p = 0.00004, F (1,20) = 17.88, a p = 0.038, b. p = 0.029, c. p = 0.00018, D TNFα, ND p = 0.019 F (1,20) = 6.48, D p = 0.00348, F (1,20) = 10.96, a. p = 0.0027, b. p = 0.0008. E IL1β D p = 0.019 F (1,20) = 7.55, D p = 0.94, a. p = 0.00027, b. p = 0.0128. F PCA blood, ND p = 0.092, D p = 0.816, a. p<0.01.

Figure 1 shows the results, with and without differentiating among the subjects on the basis of their coping style. With the risk-taking or coping style omitted from the analysis, three of the 5 mRNAs showed significant changes in expression 24 hours after an inflammatory LPS challenge. In the case of enolase (Fig 1B), this involved down-regulation; in the case of the two cytokines (Fig 1D & E), it involved up-regulation. Expression of GAPDH and CR were unaffected by the challenge (Fig. 1A and C), although the control fish showed strikingly greater variation than the experimental group. Indeed, high variability in the control group is typical of all the mRNAs studied.

The picture looks very different, however, once coping style is incorporated as an explanatory variable in the analysis. First of all, significant differences between proactive and reactive fish were found in the control group for all mRNAs, with lower (and less variable) levels in proactive fish. It is worth noting that an equivalent difference under control conditions was also observed in other tissues analysed from the same individuals (gills, liver and head kidney, data not shown). Therefore coping style influenced gene expression in carp prior to the immune challenge and explains much of the marked variability among the control fish. Secondly, in the experimental group, for four of the mRNAs, proactive and reactive fish showed significantly different responses to challenge. For GAPDH, enolase and CR (Fig. 1A–C respectively), mRNA expression was significantly down-regulated in reactive fish and up-regulated in proactive fish; as a consequence GAPDH and CR mRNA abundance in the two phenotypes converged after LPS challenge. In the case of TNFα mRNA (Fig. 1D) up-regulation is seen in proactive fish only.

The shifts in gene expression reported here are associated with equivalent changes at the whole organism level. For example, Figure 1F shows mean values for a composite score of blood condition, derived by combining haematocrit, haemoglobin concentration and red blood cell count using principal components analysis. Leaving behavioural phenotype out of the analysis, we see no significant effect of LPS injection; additionally the control group showed large variance. Phenotype alone did not have a significant effect, but there was a significant interaction between phenotype and treatment. In the control condition, reactive fish had somewhat higher scores than proactive fish. Proactive fish responded to LPS treatment with a slight increase, but timid fish responded with a dramatic decrease. This is comparable to the pattern of change shown by enolase and GADPH.

## Discussion

A first conclusion from the work described is that proactive and reactive carp maintain different levels of mRNAs under control conditions. Consequently, including coping style in our analysis has reduced unexplained variation in our control group and so has greatly increased the interpretative value of the experimental dataset. A second conclusion is that, for 80% of the genes whose expression we studied, proactive and reactive fish showed quite distinct and sometimes diametrically opposite patterns of change in response to a LPS-induced inflammatory challenge. Thus, the apparent lack of effect of this challenge on expression of mRNAs for GAPDH and CR is clearly an artefact of combining data from proactive and reactive fish, in which expression changes significantly but in opposite directions. It is important to note that the position would not have been clarified by simply increasing the number of individuals used in the experiment. As another example, the significant down-regulation of enolase mRNA and up-regulation of TNFα mRNA following LPS challenge in the combined data are the result of changes in one group of fish only. In this case, ignoring coping style gives misleading, oversimplified results. Only for IL1β do the pooled data represent equivalent changes in gene expression in both groups, though even this fails to reflect the strikingly lower variability in gene expression in proactive fish compared to reactive ones.

The regulation of proinflammatory gene expression in fish has received much attention in recent years, where major cytokines involved in the development of inflammation including TNFα and IL1β have been well characterised at the level of gene expression in a number of different fish models including the carp [22]–[25]. Interestingly a clear picture as to the regulation of these cytokines by LPS challenge has not yet emerged, due to the individual variation observed in such gene expression studies. Our data show that increases in TNFα mRNA expression pertain to one group of fish (proactive) and the effects are hidden by the high level of constitutive expression observed in reactive fish. These data directly contribute to understanding proinflammatory cytokine biology in fish suggesting that fundamental differences in cytokine regulation exist in fish with different coping styles.

In our study therefore, screening *a priori* for coping style has given us a clearer and richer picture of the effects of LPS challenge at the transcriptome level and has prevented us from drawing false conclusions. Since coping styles have been found in many animal species, this is of considerable general significance; we conclude that similar beneficial effects of including coping style as an explanatory variable in gene expression studies are likely to be widespread.

A common and sensible first step in studies aimed at linking changes in gene expression to particular environmental challenges has been to concentrate on specific strains of a few model species, with strict control of environmental conditions and using pooled data and/or average values. In this way variability is treated as background noise and minimised, so that broad effects can be exposed. This approach is not possible when, for many good reasons, the target organism comes from a natural population, rather than being a model species in which strains of known genetic identity are available. In such cases, adaptive inherited variability (which in the case of animals is likely to include differences in coping style) may well confound interpretation of pooled results.

When variability in gene expression is specifically addressed, significant inter-individual and inter-population variation has been observed. Indeed, natural variation in gene expression between individuals within a population may be higher than variation between populations [26], [27]. The reasons behind such high inter-individual variation is unknown [3]–[6]. We suggest that at least part of the variation reflects differences in coping style, maintained within populations because the phenotype is subjected to disruptive selection [28]. We therefore strongly recommend that, wherever possible, coping style be included as an additional variable in studies of differential gene expression using natural populations. Behavioural biologists have developed an array of easily-deployed techniques for screening for predictors of coping styles that can readily be adapted for use on a variety of species.

The use of molecular tools to characterise changes in gene expression in response to environmental challenge in natural populations has become extensive. In particular, the use genomic technologies such as microarray analysis are increasing in popularity, allowing such questions to be addresses in a wide range of species [27], [29], [30] and, indeed, such studies are now widespread across the animal world. From our data we conclude that, where natural populations are used in such studies, failure to include coping style as an explanatory variable may limit the interpretation of results. Conversely, combining behavioural screening for coping strategy with gene expression studies provides a powerful approach to exploring the link between gene expression and adaptive change in natural populations.

## Materials and Methods

### Broad research strategy

We conducted a study of the effects on gene expression of inflammatory challenge with bacterial lipopolysaccharide (LPS) in common carp (*Cyprinus carpio*). Prior to experimental manipulation fish were screened for coping style. Screened fish were held under normal aquarium conditions over a period of 6 weeks and then challenged with the inflammatory agent. Various measures of blood function were recorded. The abundance of 5 mRNAs were analysed in the brain of the carp under normal and challenge conditions. These mRNAs (chosen to cover a range of responses; metabolic, stress and immune) were GAPDH, enolase, cortisol receptor and 2 pro-inflammatory cytokines, tumor necrosis factor-alpha (TNFα) and interleukin 1-beta (IL1β). This study was carried out in full accordance with all national, Polish government, and local ethical committee guidelines, for the use of animals in research.

### Screening for coping style

As in a many previous studies in fish, a behavioural predictor of coping style, risk was assessed by screening the rate at which individuals explored an unfamiliar, potentially-dangerous environment [16]. This is known to be a consistent individual trait in carp that is predictive of behaviour in other contexts and of metabolic and stress physiology. Since carp are strongly schooling fish and become highly stressed in isolation, they were tested in groups.

One-year old carp (mean weight c. 24 g) were harvested from winter ponds at the Polish Academy of Sciences' Institute of Ichthyobiology and Aquaculture, Cieszyn, Poland in April 2006, treated for infection and stocked in groups of 70 in 35 liter tanks with recirculated water. After settling for 1 week, fish were deprived of food for at least 12 h and tested for coping style, risk-taking in a novel environment, as follows. 10 randomly-selected fish were removed from their holding tank in covered buckets and tipped gently into a setting area at one end of a well lit tank (1.5×1 m). The settling area comprised of a covered circular opaque black compartment (diameter 50 cm) fitted at the base with a closeable exit tube (diameter 10 cm). A covered area at the opposite end of the main tank incorporating a closable gate was installed in the fish collection area.

The fish were allowed to settle for 5 min, during which food extract (prepared by soaking food pellets in water) was gently tipped into the test compartment, just in front of the exit tube. The cover of the exit tube was then removed and a two-phase observation period initiated. After the first 3 carp had emerged from the settling area, or after a period of 10 minutes if fewer than three fish emerged during this period, the exit tube was closed and the fish that had emerged gently edged into the fish holding compartment and the gate closed. These fish were classified as risk taking, proactive individuals. A second small amount of feed extract was added in front of the exit tube, which was then opened and a second recording period started, during which a further four fish were allowed to emerge and the exit tube was closed again. These fish were classified as of intermediate coping strategy. The 3 fish remaining in the starting shelter were confined in the shelter by replacing the lid; these fish were classified as risk-avoiding, reactive fish. If fewer than four intermediate fish emerged during 15 minutes of observation, all the remaining fish were classified as reactive. After screening, reactive and proactive fish were given batch marks using Alcian blue dye and the intermediate fish were discarded.

### Inflammatory challenge

Screened fish were held in groups of 20 (10 reactive and 10 proactive) in 35 l tanks under normal aquarium (7–8 mg O_2_/l and 20°C) conditions fed daily with 2 mm diameter pellets (Aller, Danmark) with a ration of 2% fish biomass per day. After 6 weeks, 6 proactive and 6 reactive fish were challenged with the inflammatory agent, LPS (lipopolysaccharide *E.coli*, Sigma). A further 6 fish of each coping style were give a sham injection (0.9%NaCl, buffered). After c. 20 hours fish were anesthetized (*Propiscine*), killed and weighed. Blood was extracted from the caudal vein and hemoglobin concentration estimated using Drabkin's method, percentage red blood cell volume measured by hematocrit and red blood cells counted in a Burker chamber using light microscopy. These were combined using Principal Components Analysis, the first component of which accounted for 73% of total variance, had high positive loadings for all three variables and was used as an integrated index of blood function. Material collected from the gills, brain, head kidney and liver was frozen on dry ice and transported to the Department of Animal Physiology of the Autonomous University of Barcelona, Spain. Total RNA was extracted from the tissues using TriReagent (Molecular Research Center) following the manufacturer's instructions, and verified for quantity and integrity by denaturing electrophoresis gel for RNA.

### Quantitative PCR

In order to measure gene expression in individual fish; 4 µg of total RNA was taken from individual brain samples to synthesize cDNA with SuperScript III RNase Transcriptase (Invitrogen) and oligo-dT primer (Promega). cDNA was diluted 1∶100 for the amplification of selected genes and 1∶1000 for 18S, and used as a template with primers designed for Q-PCR (Table 1).

**Table 1:** Specific primer sets for QPCR.

Gene	Primer	Tm (°C)	Sequence	Size
S18	For	60	5′-CGA GCA ATA ACA GGT CTG TG-3′	212
	Rev		5′-GGG CAG GGA CTT AAT CAA-3′	
CR	For	60	5′-CCA GCA AGA ACT GGC AAC GA-3′	150
	Rev		5′-TGA TGA TCT CCG CCA GCA TT-3′	
GAPDH	For	60	5′-AGG CGG CAA GCT GGT CAT T-3′	189
	Rev		5′-GCA CTG GGG GCA GAG ATG A-3′	
ENO	For	57	5′- ATC CAG TCC AGT CCA TCG AGG ATC C-3′	167
	Rev		5′- GAG GAG CAG GCA GTT ACA GG-3′	
IL	For	58	5′- AAG GAG GCC AGT GGC TCT GT-3′	168
	Rev		5′- CCT GAA GAA GAG GAG GCT GTC-3′	
TNF	For	58	5′- GCT GTC TGC TTC ACG CTC AA-3′	174
	Rev		5′- CCT TGG AAG TGA CAT TTG CTT TT-3′	

Wells (20 µl final volume) contained 10 µl of iQ™ SYBR Green Supermix (Bio-Rad), 500 nM concentration of forward and reverse primers and 5 µl of cDNA. Controls lacking cDNA and controls containing RNA were included. Reactions were run in a MyiQ thermocycler (BioRad) under the following protocol: 5 min initial denaturation at 95°C, followed by 40 cycles of 10 sec denaturation at 95°C and 30 sec at annealing temperatures, and a final melting curve of 81 cycles (from 55°C to 95°C). All samples were run in triplicate and fluorescence was measured at the end of every extension step. C_T_ (threshold cycle) values for each sample were expressed as “fold differences”, calculated relative to control diet and normalized for each gene against those obtained for 18S. Transcripts were sequenced to ensure amplification was specific: products were visualized under UV light in a 1% agarose gel containing 1 mg/ml ethidium bromide, purified using MiliElutegel purification system (Quiagen), cloned into PGEM-T Easy Vector (Promega) by T/A cloning and transfected into competent Escherichia coli JM 109 cells (Promega). Plasmid DNA was isolated by Nucleospin Quickpure (Marcherey Nagel), digested with EcoRI (Promega) and sequenced with T7 primer.
